# Polymorphic Alpha-Synuclein Oligomers: Characterization and Differential Detection with Novel Corresponding Antibodies

**DOI:** 10.1007/s12035-023-03211-3

**Published:** 2023-01-28

**Authors:** Kenya Moore, Urmi Sengupta, Nicha Puangmalai, Nemil Bhatt, Rakez Kayed

**Affiliations:** 1 Mitchell Center for Neurodegenerative Disease, University of Texas Medical Branch, Galveston, TX USA; 2 Department of Neurology, Neuroscience and Cell Biology, Medical Research Building Room 10.138C, University of Texas Medical Branch, 301 University Blvd, Galveston, TX 77555-1045 USA

**Keywords:** Alpha-synuclein, Oligomers, Neurotoxicity, Amyloid, Monoclonal antibodies, Protein aggregation

## Abstract

The pathological hallmark of many neurodegenerative diseases is the accumulation of characteristic proteinaceous aggregates. Parkinson’s disease and dementia with Lewy bodies can be characterized as synucleinopathies due to the abnormal accumulation of the protein alpha-synuclein (α-Syn). Studies have shown amyloidogenic proteins such as α-Syn and tau can exist as polymorphic aggregates, a theory widely studied mostly in their fibrillar morphology. It is now well understood that an intermediate state of aggregates, oligomers, are the most toxic species. We have shown α-Syn, when modified by different physiological inducers, result in distinct oligomeric conformations of α-Syn. Polymorphic α-Syn oligomers exhibit distinct properties such as aggregate size, conformation, and differentially interact with tau. In this study, we confirm α-Syn oligomeric polymorphs furthermore using in-house novel α-Syn toxic conformation monoclonal antibodies (SynTCs). It is unclear the biological relevance of α-Syn oligomeric polymorphisms. Utilizing a combination of biochemical, biophysical, and cell-based assays, we characterize α-Syn oligomeric polymorphs. We found α-Syn oligomeric polymorphs exhibit distinct immunoreactivity and SynTCs exhibit differential selectivity and binding affinity for α-Syn species. Isothermal titration calorimetry experiments suggest distinct α-Syn:SynTC binding enthalpies in a species-specific manner. Additionally, we found SynTCs differentially reduce α-Syn oligomeric polymorph-mediated neurotoxicity and propagation in primary cortical neurons in a polymorph-specific manner. These studies demonstrate the biological significance of polymorphic α-Syn oligomers along with the importance of polymorph-specific antibodies that target toxic α-Syn aggregates. Monoclonal antibodies that can target the conformational heterogeneity of α-Syn oligomeric species and reduce their mediated toxicity have promising immunotherapeutic potential.

**Supplementary Information:**

The online version contains supplementary material available at 10.1007/s12035-023-03211-3.

## Introduction

Alpha-synuclein (α-Syn) is a 140-amino acid, presynaptic protein whose physiological function has been linked to neurotransmitter release and regulation of synaptic vesicle trafficking [1–4]. α-Syn is encoded by the SNCA gene and is abundantly expressed in the brain [3]. Multiple studies have shown in the presence of factors such as oxidative stress, impaired cellular degradation, and genetic mutations, α-Syn aggregates into larger, toxic structures [5–9]. α-Syn is often referred to as an amyloidogenic protein, a soluble monomeric precursor that undergoes conformational changes, resulting in the formation of fibrils and ultimately amyloid aggregates called Lewy bodies [10–14].

Several studies have shown that amyloid aggregates, specifically fibrils, exhibit polymorphism [15, 16]. α-Syn fibrils exhibit polymorphism at the molecular level and buffers utilized for growth conditions have been shown to cause distinct morphology and biological activity of α-Syn fibrils [17, 18]. However, accumulating evidence, both in vitro and in vivo, has shown that an intermediate state of aggregates, oligomers, are the most potent neurotoxic species [19, 20]. The implication of polymorphic α-Syn oligomers in synucleinopathy pathology and disease pathogenesis is not yet fully investigated. Our lab recently demonstrated that α-Syn can form polymorphic oligomers with distinct biochemical and biophysical properties in vitro that may have pathological significance [21]. This supports previous reports of growth conditions and physiological inducers triggering distinct conformational and aggregation states of α-Syn. [21–25]. We hypothesize heterogeneous amyloid aggregates contribute to disease variability observed in synucleinopathies such as Parkinson’s disease (PD) and dementia with Lewy Bodies (DLB). Conformation-specific antibodies that can recognize and bind distinct α-Syn oligomers have the potential to halt or reduce α-Syn aggregation and offer novel insight into polymorphic α-Syn oligomer-mediated toxicity and biological relevance [26, 27].

α-Syn oligomers were prepared and modified by three different physiologically relevant inducers/buffers including artificial cerebrospinal fluid (aCSF), docosahexaenoic acid (DHA), and dopamine. Elevated levels of α-Syn oligomers have been observed in the brains of patients with both PD and DLB [28]. Additionally, Groveman et al. demonstrated pathogenic disease-associated forms of α-synuclein seeding activity are present in cerebrospinal fluid using a novel assay [29]. Polyunsaturated fatty acids, such as DHA, and their peroxidation byproducts have been shown to induce oligomerization of α-Syn [30]. Interestingly, propanoylated lysine, a product of DHA oxidation, was found increased in neuronal differentiated human neuroblastoma SH-SY5Y cells overexpressing α-synuclein [31]. The loss of dopaminergic neurons is a well-known characteristic of Parkinson’s disease (PD). In addition, dopamine has been shown to induce soluble α-Syn oligomers and preformed fibrils which ultimately lead to nigrostriatal degeneration in a PD mouse model [32, 33]. Based on these novel findings, we began our investigation into physiologically relevant α-Syn oligomeric polymorphs.

The best therapeutic approach to bind and reduce the burden of α-Syn aggregates is still undetermined and if this reduction will halt neurotoxicity is unknown [34]. Utilizing monoclonal antibodies to therapeutically target α-Syn aggregates allows for pharmacologic control and has been shown to be successful in other neurodegenerative diseases [35, 36]. Multiple antibodies have been developed to target aggregated α-Syn; however, many have been unsuccessful [37–39].

**Fig. 1:**
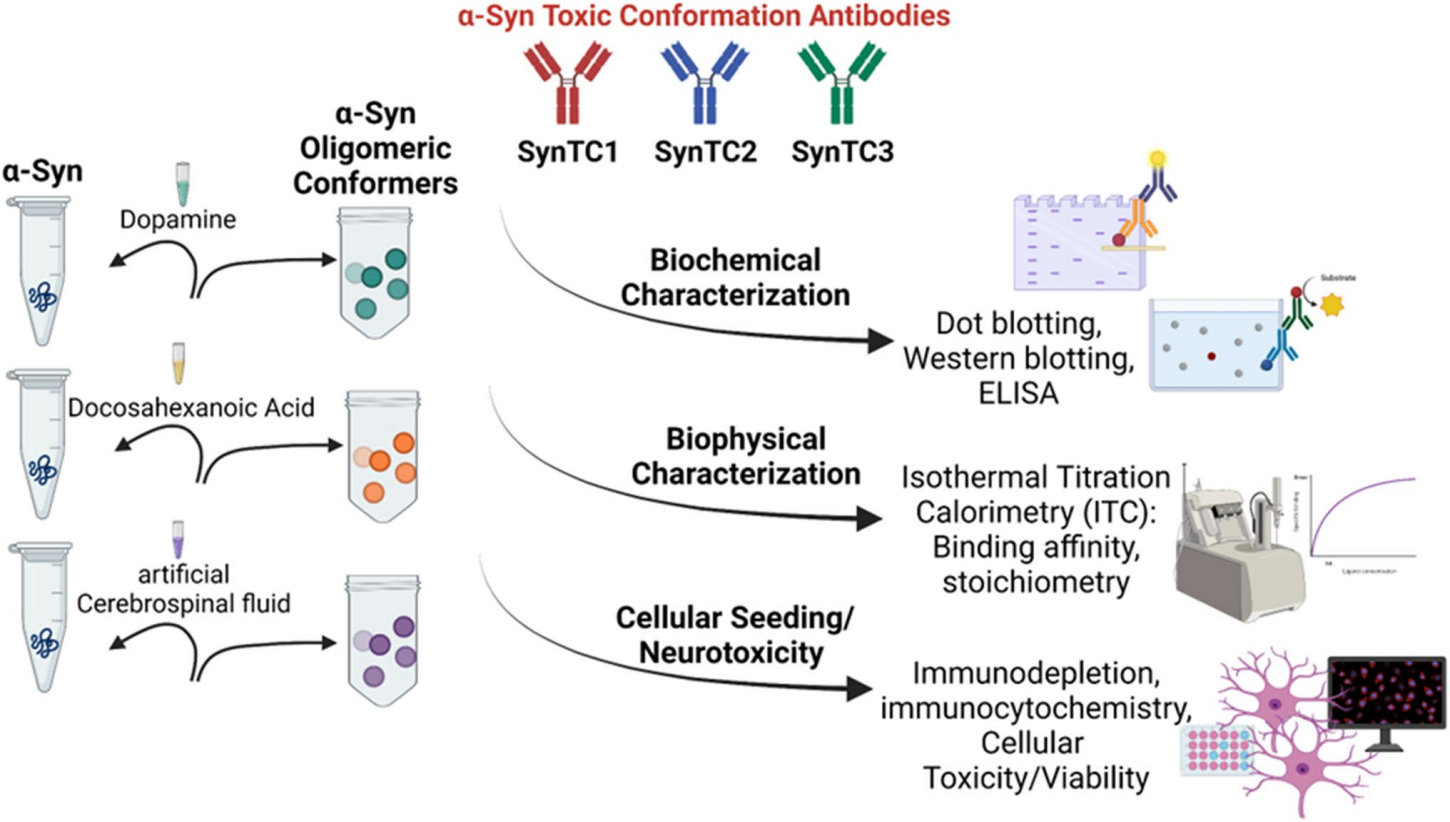
Schematic of experimental overview. Three different α-Syn oligomeric polymorphs were systematically characterized biochemically and biophysically and evaluated by cellular spreading. In addition to traditional methods, we used 3 novel α-Syn toxic conformation antibodies

In this study, we utilized standard biochemical and biophysical methods to investigate immunological properties of three distinct α-Syn oligomeric conformations with well-established assays and our α-Syn Toxic Conformation monoclonal antibodies (SynTCs) (Fig. 1). For an antibody to have therapeutic potential, it must exhibit selectivity and affinity for aggregated forms of α-Syn and reduce α-Syn cellular internalization and mediated neurotoxicity. Here, we demonstrate the distinct immunoreactivity and seeding propensity of each α-Syn oligomeric polymorph and the ability of SynTCs to interfere with their mediated toxicity. When α-Syn oligomers are immunodepleted by SynTCs, cells exhibit differential reduction in toxicity and endogenous aggregation of α-Syn aggregates.

## Methods

### Generation of α-Syn monomers, oligomers, and fibrils

Recombinant α-Syn monomer, oligomers, and fibrils were prepared following our published methods [21, 40, 41].

### Generation of SynTCs

Briefly, the cell lines for the antibodies were developed utilizing α-Syn oligomer antigen prepared in our lab (Genscript Biotech, NJ, USA). Several cell lines were evaluated for their immunoreactivity using different preparations of α-Syn oligomers and α-Syn monomer by dot blot, filter trap assay, and enzyme-linked immunosorbent assay (ELISA). Finally, the three SynTC clones were selected for production.

### SynTC Screening

#### Indirect ELISA

Anti-syn oligomer antibody response was determined by screening serial dilutions of animal sera using an Indirect enzyme-linked immunosorbent assay (ELISA) as previously published [42, 43]. Briefly, 96-well plates (Nunc Immobilizer, Amino Plates and Modules, 436,006, Thermo Fisher Scientific) were previously coated with 1 μl α-Syn oligomers, Aβ oligomers, or tau oligomers in 50 μl of 1 × PBS, pH 7.4, as coating buffer. After washing three times with TBS-T, plates were blocked for 2 h at room temperature with 10% nonfat milk in TBS-T. Plates were then washed three times with TBS-T and probed with 100 μl of primary antibodies for 1 h at room temperature (RT): commercial antibodies, LB509 (1:5000;Abcam 27,766), Syn211 (1:5000;Abcam 80,627)), sequence-specific α-Syn antibodies Syn 33 (1:1000;SigmaAldrich ABN2265), MJFR (1:1000;Abcam 209,538), and F8H7 (1:1000). Plates were then washed three times with TBS-T and incubated with 100 μl of HRP-conjugated anti-rabbit or anti-mouse IgG, diluted 1:10,000 in 5% nonfat milk in TBS-T, for 1 h at room temperature. Plates were washed three times with TBS-T and developed with 3,3,5,5-tetramethylbenzidine (S1599, Dako). The reaction was stopped using 100 μl of 1 m HCl, and absorbance was read at 450 nm using a POLARstar OMEGA plate reader. All experiments were performed in triplicate.

#### Dot Blotting

Dot blot was also used to test SynTC specificity. Each strip had seven protein dots: dot #1 (α-Syn monomer), dots #2–4 (α-Syn oligomers from different preparations), dot #5 (α-Syn fibrils), dot #6 (tau oligomer), and dot #7 (Aβ oligomer). One microliter of each sample was dotted on nitrocellulose membrane. Next, membranes were blocked with 10% nonfat milk in TBS-T overnight at 4 °C. After blocking, membranes were probed with previously listed antibodies in 5% nonfat milk for 1 h at RT. This was followed by incubation with HRP-conjugated IgG anti-mouse or anti-rabbit (1:6000, GE Healthcare) secondary antibody to detect primary antibodies. ECL plus (GE Healthcare) was used for signal detection [44].

#### Western Blotting

One microgram per microliter of each sample (α-Syn monomer, α-Syn oligomers, α-Syn fibrils, tau oligomer, and Aβ oligomer) was resolved on a precast NuPAGE 4–12% BisTris gel for SDS-PAGE (NP0335BOX, Invitrogen) and transferred to nitrocellulose membranes. Next, membranes were blocked with 10% nonfat milk in TBS-T overnight at 4 °C. After blocking, membranes were probed with previously listed antibodies, diluted in 5% nonfat milk for 1 h at RT. Membranes were then incubated with HRP-conjugated IgG anti-mouse (1:6000, GE Healthcare) secondary antibody to detect α-Syn antibodies. ECL plus (GE Healthcare) was used for signal detection. Finally, the selected clones (SynTCs) were tested using human and mouse brains [44–47].

### Biochemical Characterization of α-Syn Oligomeric Strains

#### Indirect ELISA

ELISA plates were coated with 1 µg/well of α-Syn monomer, α-Syn oligomeric polymorphs, and tau oligomer. 0.1 M sodium bicarbonate, pH 9.6, was used as a coating buffer followed by overnight incubation with primary antibodies: SynTC1 (1:4000), SynTC2 (1:1000), SynTC3 (1:1000), and total α-Syn commercial antibody LB509 (1:5000; Abcam 27,766) at 4 °C. Plates were then washed three times with TBS-T and incubated with 100 μl of HRP-conjugated anti-mouse IgG, diluted in 5% nonfat milk in TBS-T, for 1 h at room temperature. Plates were washed three times with TBS-T and developed with 3,3,5,5-tetramethylbenzidine (S1599, Dako). The reaction was stopped using 100 μl of 1 m HCl, and absorbance was read at 450 nm using a POLARstar OMEGA plate reader. All experiments were performed in triplicate [42, 43].

#### Dot Blotting

One microliter of each sample, dot #1 (α-Syn monomer), dots #2–4 (α-Syn oligomeric polymorphs), dot #5 (α-Syn fibrils), dot #6 (tau oligomers), and dot #7/#8 (Aβ40/42 oligomers) was dotted on nitrocellulose membrane and let dry for 1 h at RT. Next, membranes were blocked with 10% nonfat milk in TBS-T overnight at 4 °C. After blocking, membranes were probed with primary antibodies SynTC1 (1:4000), SynTC2 (1:1000), SynTC3 (1:1000), and total α-Syn commercial antibody LB509 (1:5000; Abcam 27,766) in 5% nonfat milk for 1 h at RT followed by incubation with HRP-conjugated IgG anti-mouse (1:6000, GE Healthcare) secondary antibody to detect α-Syn antibodies. ECL plus (GE Healthcare) was used for signal detection [44].

#### Western Blotting

For western blotting, 1 μg of each sample (α-Syn monomer, α-Syn oligomeric polymorphs, (α-Syn fibril, tau oligomer, and Aβ40/42 oligomers) were loaded on precast NuPAGE 4–12% Bis–Tris gels (Invitrogen) for SDS-PAGE analysis. Gels were subsequently transferred onto nitrocellulose membranes. Membranes were blocked with 10% nonfat dry milk at 4 °C overnight. This was followed by incubation with primary antibody followed by secondary antibody incubation. After antibody binding, the membrane is incubated with chemiluminescent substrate and imaged. Primary antibodies for each experiment include SynTC1 (1:4000), SynTC2 (1:1000), SynTC3 (1:1000), and total α-Syn commercial antibody LB509 (1:5000; Abcam 27,766). HRP-conjugated, anti-mouse IgG (1:6000, GE Healthcare) was used to detect each SynTC and LB509. ECL plus (GE Healthcare) was used to visualize the bands [44–47].

### Proteolytic Digestion of α-Syn Oligomers by Proteinase K Enzyme

Different oligomer preparations of α-Syn (10–12 μg) were treated with different concentrations of proteinase K enzyme (Sigma) ranging from 1 to 2 μg/mL in the presence of 1 × PBS buffer and incubated at 37 °C for 30 min. At the end of incubation time, 1 × LDS sample buffer (Invitrogen) was added and heated at 95 °C for 10 min. Samples were immediately transferred onto ice to stop the cleavage reaction followed by loading the digestion products into 4–12% Bis–Tris precast gels (Invitrogen) for SDS-PAGE gel electrophoresis. Gels with digested samples were processed for silver staining (Pierce Silver Stain Kit, Thermo Scientific; 24,612) to visualize the fragments following the manufacturer’s instructions [21].

### Atomic Force Microscopy

SynOaCSF and fibrillar α-Syn were analyzed by AFM using a non-contact tapping method with a Multimode 8 AFM machine (Bruker, Billerica MA). Briefly, 3–4 μl of each sample was applied onto a fresh-cleaved mica surface and allowed to adsorb at RT overnight. Mica was then washed with 200 μl of deionized water and air-dried. Images were taken from 5 different areas on the mica surface [21].

### Isothermal Titration Calorimetry (ITC)

ITC measurements were made using a MicroCal PEAQ-ITC [48]. 8 μM of either SynTC1, 2, or 3 was titrated into 2 μM α-synuclein monomer, unmodified oligomer, or fibril using an initial 0.4-μL injection followed by 2-μL injections at intervals of 2 min, with a stirring rate of 1000 rpm. The temperature was maintained at 25 °C. Protein samples were prepared in phosphate buffer at a pH of 7.4. Titrations of ligand into buffer were measured and used for background subtraction before fitting the data. A one set of sites binding model was used for all experiments and binding curves were fit with a Gaussian nonlinear regression model on Prism 9.4 (GraphPad Software) [49–51]. Data at each step of analysis is provided in supplementary figures (Supp. Figs. 5, 6, and 7).

### Cell Treatment with α-Syn Oligomers

Human neuroblastoma, SH-SY5Y, cells were cultured in high glucose Dulbecco’s modified Eagle’s medium (DMEM, Gibco) supplemented 10% fetal bovine serum (Gibco) and 1% penicillin/streptomycin (Gibco). α-Syn oligomeric polymorphs (0.5 μM), generated as previously published [21], were incubated with each SynTC (2 μM) for 30 min at RT. Cells were then exposed to SynO polymorphs or SynTC-immunodepleted SynO polymorphs for 24 h.

### Primary Cortical Neuron Culture and Treatment

C57BL/6 transgenic mice expressing human α-Syn (Jackson Laboratory, 017,682) were used for primary cortical neuron isolation. Primary cortical neuronal cells from mice during embryonic days 16–18 were isolated using Accutase solution (Sigma, A6964) and maintained as previously published [21]. Briefly, neuronal cells were plated on poly-D-lysine-coated glass coverslips (Corning, Inc.) at a density of 2 × 105 cells/mL in a 24-well plate containing neurobasal medium (Gibco, 12,348,017) supplemented with 2% B-27, 0.5 mM GlutaMax (Gibco, 35,050–061), 10,000 units/mL penicillin, 10,000 μg/mL streptomycin, and 25 μg/mL amphotericin B supplement. Media changes were performed every 3–5 days by replacing 50% culture media with fresh media. Cells were grown for 10–13 days in vitro (DIV) before experiments. Primary cortical neurons were grown on coverslips in 24-well plates. α-Syn oligomeric polymorphs (0.5 μM), generated as previously published [21], were incubated with each SynTC (2 μM) for 30 min at RT. Primary cortical neurons were then exposed for 24 h [44, 45]. The procedures involving experimentation on animal subjects are done in accordance with UTMB’s guidelines.

### Immunocytochemistry and Image Analysis

Following the 24-h incubation, cells were washed 3 times with 1 × PBS and fixed with 4% formaldehyde solution for 15 min at RT. Cells were then washed 3 times with 1 × PBS followed by permeabilizing with 0.25% Triton X-100 in PBS for 10 min at RT. Cells were blocked in 5% goat serum for 30 min at RT and incubated with primary antibodies: βIIITubulin (1:1000; Abcam78078), Syn10842 (1:1000; ThermFisher 10,842–1-AP), and total α-Syn antibody LB509 (1:5000; Abcam 27,766) at 4 °C overnight. The next day, cells were washed and incubated with Alexa-conjugated secondary antibodies (1:000; Life Technologies) for 1 h at RT in the dark. After three washes, cells were mounted with Prolong Gold antifade reagent with DAPI. Each treatment condition was performed in 3 replicates and were randomly imaged at five different regions of interest. Images were captured with a Keyence BZ-800 Microscope and analyzed using BZ-X Analyzer. A Nikon 60 × objective was used for image acquisition. To eliminate species cross-reactivity, fluorescent intensity of total α-Syn was quantified by total α-Syn anti-rabbit polyclonal, Syn10842 (1:1000; Thermo Fisher 10,842–1-AP). All images were analyzed by ImageJ (NIH) software. Statistical significance is measured by using two-way ANOVA with Bonferroni post hoc analysis. **p < 0.01, ****p < 0.0001. Scale bar 10 μm.

### Cell Toxicity and Viability Assays

Cytotoxicity was determined by measuring lactate dehydrogenase (LDH) release using Cytotoxicity Detection kit PLUS (Roche, 04,744,926,001), and cell viability was measured by CellTiter 96® Aqueous Non-Radioactive Cell Proliferation Assay (MTT) (Promega, G5421) following manufacturers’ instructions as previously described. In brief, following strain and immunodepletion treatment for 24 h, cells were assayed with LDH or MTT for cytotoxicity and cell viability assays respectively. For both assays, absorbance was measured at 490 nm with a Polar Star Omega plate reader (BMG Labtech). Each experimental condition was performed in triplicates in three different independent assays. For the MTS assay, the percentage of viable cells was calculated as ((ODtreated -ODuntreated control)/ODuntreated control) × 100. For LDH assay, the percentage of affected cells was calculated following the formula provided by the manufacturer.

### Statistical Analysis

All experiments were repeated at least three times. Statistical analyses were performed using Prism 9.4 (GraphPad Software, Inc., San Diego, CA). All values were calculated as mean and standard deviation. For cytotoxicity and fluorescent intensity, two-way analysis of variance (ANOVA) with Bonferroni post hoc analysis was conducted. Additional details are mentioned in figure legends. 

## Results

### Biochemical Characterization of α-Syn Oligomeric Strains/SynTCs

**Fig. 2:**
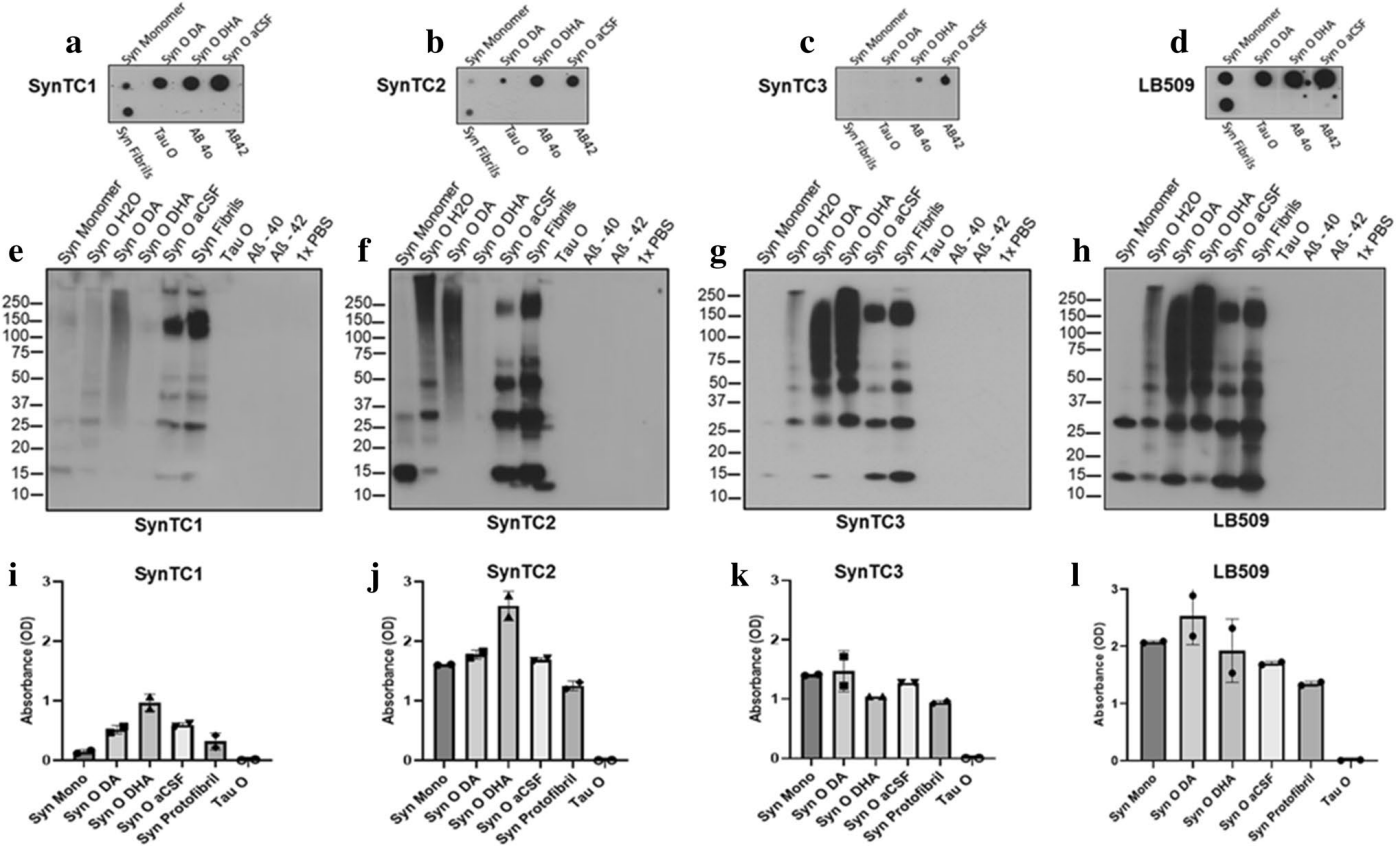
Biochemical characterization of α-Syn oligomeric polymorphs. α-Syn oligomeric polymorphs were characterized by dot blotting, quantification provided in supplementary Fig. 1 (top), western blotting (middle), and indirect enzyme-linked immunosorbent assay (ELISA) (bottom). α-Syn monomer, different α-Syn oligomer preparations, α-Syn fibrils, and amyloidogenic proteins, tau and amyloid β, were characterized with primary antibodies: SynTC1 (**a**, **e**, **i**), SynTC2 (**b**, **f**, **j**), SynTC3 (**c**, **g**, **k**). Results revealed the selectivity of the SynTCs for α-synuclein, confirmed by total Syn antibody, LB509 (**d**, **h**, **i**). Analyses confirm differences in immunoreactivity for α-Syn oligomeric conformers. Indirect ELISA: data represented as mean ± SD

We have previously shown how α-Syn oligomeric conformers differ by aggregate size, conformation, and hydrophobicity [21]. To further investigate if these oligomeric polymorphs have biological relevance, we first performed standard biochemical characterization utilizing dot blot (Fig. 2a–d), western blot (Fig. 2e–h), and indirect ELISA (Fig. 2i–l). To test the selectivity of our novel SynTCs, α-Syn monomer, α-Syn oligomeric polymorphs (SynODA, SynODHA, SynOaCSF), α-Syn fibrils, tau, and amyloid-β were used. Each SynTC exhibits selectivity for α-Syn species and distinct immunoreactivity for each oligomeric polymorph (Fig. 2). Dot blotting revealed distinct intensities detected by SynTCs for each α-Syn oligomeric species compared to detection by total α-Syn commercial antibody, LB509 (Fig. 2d). Dot blot quantification is provided (Supp. Figure 1). SynTC1 differentially detected all α-Syn species with the highest intensity for α-Syn oligomeric polymorphs and weakest intensity for α-Syn monomer (Fig. 2a). SynTC2 differentially detected all α-Syn species with low immunoreactivity for α-Syn monomer (Fig. 2b). SynTC3 detected SynOaCSF and SynODHA, but did not detect α-Syn monomer, SynODA, or α-Syn fibrils (Fig. 2c). Overall SynTCs selectively detect all α-Syn species with a higher selectivity for aggregated α-Syn. For more biochemical characterization, we performed western blotting, which revealed detection of different populations of aggregates among α-Syn oligomeric polymorphs. (Fig. 2e–h). SynTCs differentially detect monomeric species around 14 kDa, oligomeric species ranging from 25 to 75 kDA and higher molecular weight aggregates above 250 kDa (Fig. 2e–h). While SynTC2 strongly detected α-Syn monomer (Fig. 2f), SynTC1 (Fig. 2e) and SynTC3 (Fig. 2g) show the least immunoreactivity to α-Syn monomer. SynODHA did not exhibit immunoreactivity to SynTC1 or SynTC2 but exhibited strong immunoreactivity to SynTC3. All α-Syn species were detected and confirmed by total α-Syn antibody, LB509 (Fig. 2h). Next, we performed indirect ELISA and like immunoblotting results, differences in immunoreactivity were also observed (Fig. 2i–l). SynTC1 exhibited the least binding to α-Syn species (Fig. 2i) in agreement with western blotting (Fig. 2e). SynTC2 exhibited binding to α-Syn monomer (Fig. 2j) similarly shown in western blotting (Fig. 2f). α-Syn immunoreactivity detected by SynTC3 (Fig. 2k) was not as distinct among all α-Syn species compared to dot blotting (Fig. 2c).

### α-Syn Oligomeric Polymorphs Reveal Distinct Sensitivity to Proteinase K

α-Syn oligomers are different in their aggregate size, hydrophobicity, and biological properties [21]. To evaluate the conformational differences between the three oligomeric polymorphs as well as their stability as oligomers, we measured their sensitivity for proteinase K (PK) enzyme digestion. PK digestion has long been used in classifying strains of prion fibrils [52, 53]. Nevertheless, this method has been extended and widely used for identifying amyloid strains, of amyloid-β, α-Syn, and tau fibrils [54, 55]. We treated SynODA, SynODHA, and SynOaCSF with increasing concentrations of PK enzyme (0–2 μg/mL). All the digested samples were then run in SDS-PAGE followed by silver staining (Supp. Fig. 2). The pattern of fragments generated by PK digestion provides information on the stability of the oligomers, as well as its core. We observed SynODA was resistant to PK, thus indicating a stable core of these oligomers. In contrast, SynODHA and SynOaCSF were sensitive to PK showing two distinct fragmentation patterns. These results suggest each oligomeric polymorph exhibit conformational differences.

### Atomic Force Microscopy of SynOaCSF and Syn Fibrils

Due to the similar immunoreactivity exhibited by SynOaCSF and Syn Fibrils observed in biochemical studies (Fig. 2), atomic force microscopy was utilized to observe structural properties of each sample (Supp. Fig. 3). Representative AFM images of Syn O aCSF exhibit spherical structures. In contrast, Syn Fibrils exhibit protofilaments. These results confirm structural differences among SynOaCSF and Syn Fibrils [21].

### Biophysical Characterization of α-Syn Oligomeric Strains/SynTCs

**Fig. 3:**
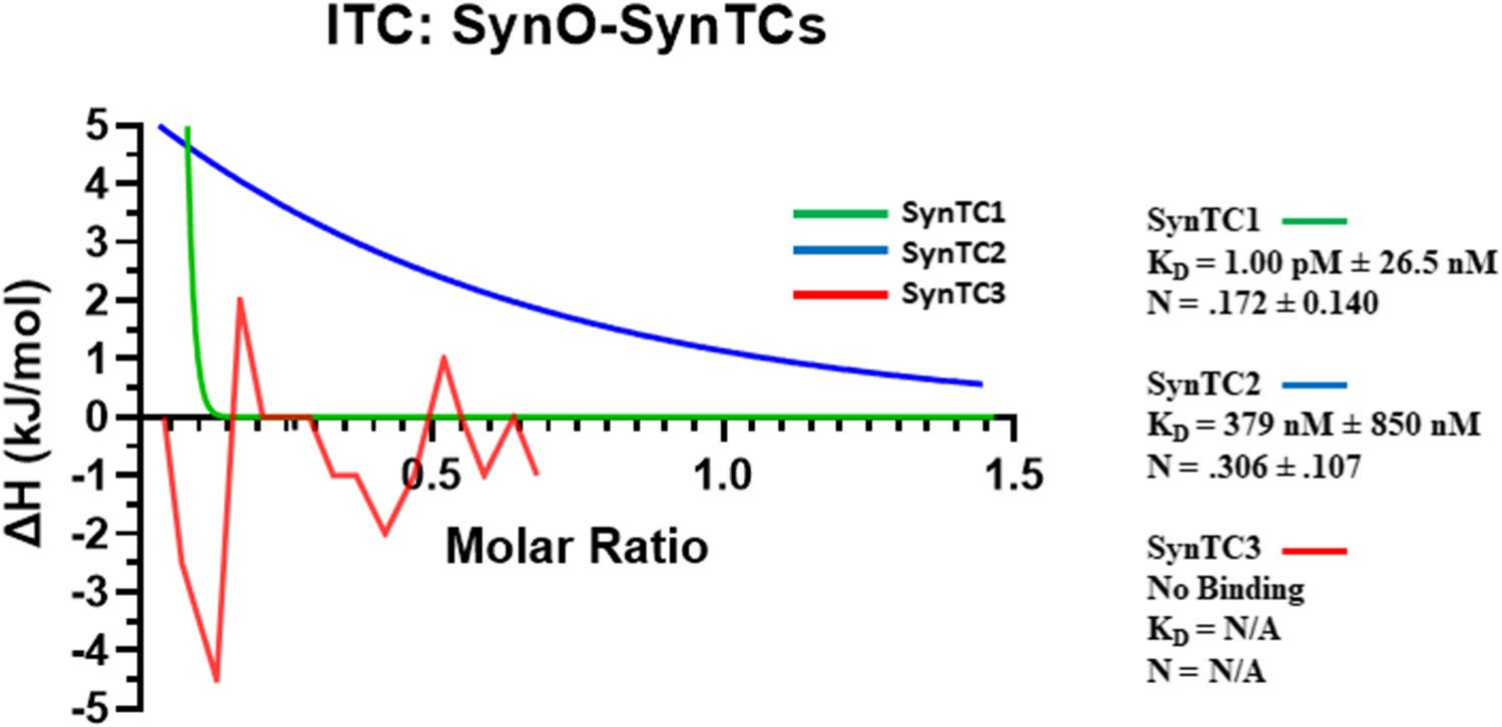
Isothermal titration calorimetry (ITC) confirms distinct binding profiles of SynTCs and α-Syn oligomers. Integrated binding curves of the isothermal titration calorimetry (ITC) experiment of 2 μM α-Syn oligomer titrated with 8 μM SynTC at 25 °C. A one set of sites binding model was used for all experiments. Binding curves were fitted with a nonlinear regression model. Thermodynamic and stoichiometric parameters obtained from the fitting of the binding curve are shown

We utilized the highly sensitive method, isothermal titration calorimetry (ITC), to quantify the binding interactions between SynTCs and oligomeric α-Syn (Fig. 3). The buffer and pH for ITC experiments were assigned and optimized based on a previously published protocol [49]. Unmodified α-Syn oligomers were utilized instead of polymorphs due to the significance of buffer sensitivity when conducting ITC [56]. Each SynTC exhibited a distinct binding profile for α-Syn oligomers. SynTC1 (green) bound oligomeric and monomeric α-Syn but not fibrillar. Results suggest SynTC1 bound α-Syn oligomer at very high affinity with a K_D_ of 1.00 pM ± 26.5 nM (Fig. 3). SynTC1 bound α-Syn monomer at a lower affinity with a K_D_ of 5.31 ± 45.1 nM (Supp. Fig. 4a). SynTC2 selectively bound α-Syn oligomer at high affinity with a K_D_ of 379 ± 850 nM (Fig. 3) and did not bind monomeric or fibrillar α-Syn (Supp. Fig. 4a, b). Lastly, SynTC3 did not bind α-Syn species. SynTC1 exhibited the highest affinity to α-Syn oligomers but also bound monomeric α-Syn at a lower affinity. SynTC2 exhibited lower affinity to oligomeric α-Syn than SynTC1 but did not bind monomeric or fibrillar α-Syn. SynTC3 does not demonstrate binding to α-Syn, which is not in agreement with previous results. These differences in binding affinity provide insight into the functionality of SynTCs and the effects of conformation-specific antibodies.

### SynTCs Differentially Reduce α-Syn Oligomeric Seeding in Primary Neurons

**Fig. 4:**
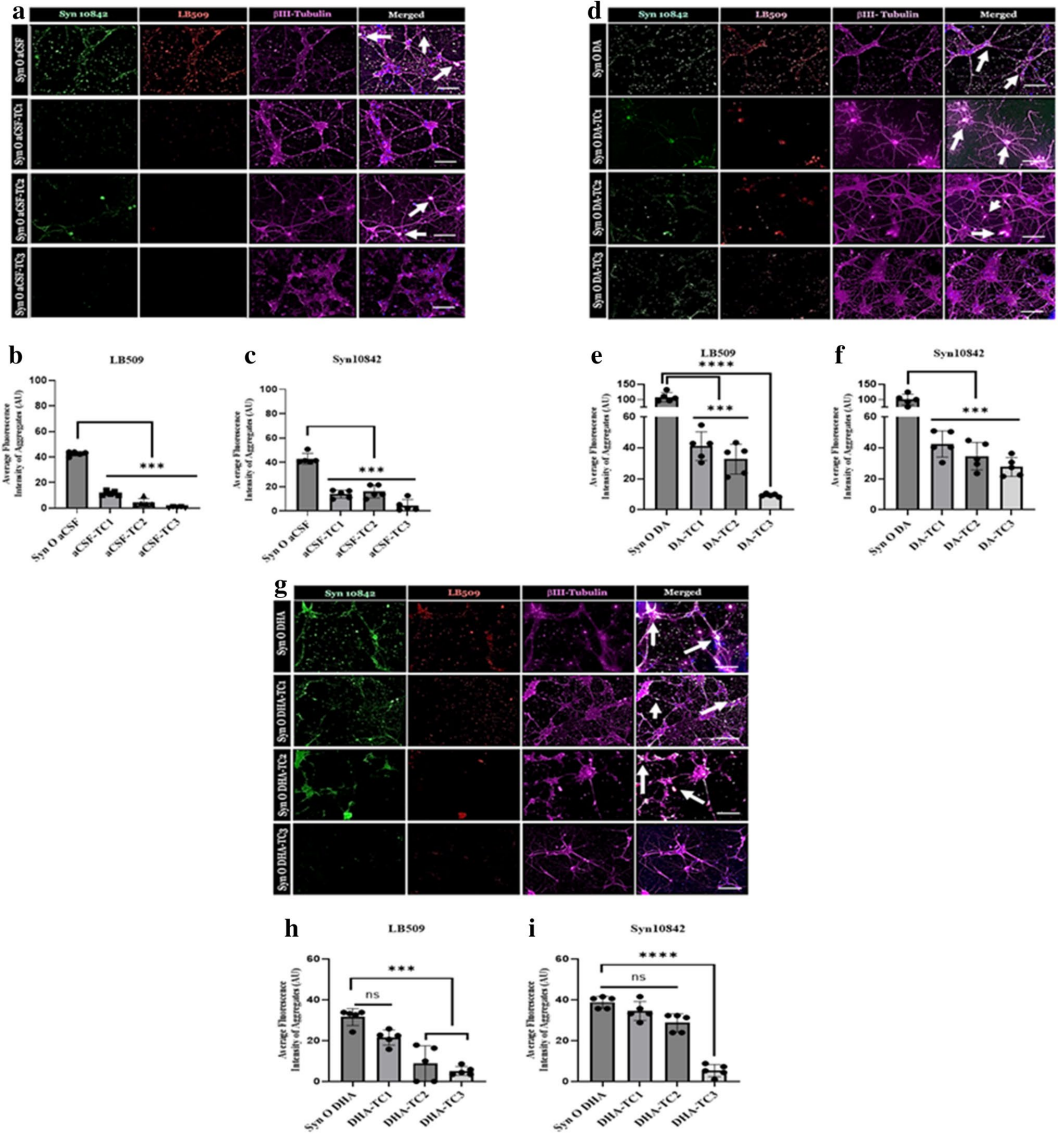
SynTC immunodepletion of α-Syn oligomeric polymorphs reduces α-Syn seeding and endogenous aggregation. Primary neurons were treated with α-Syn oligomer or α-Syn oligomer preincubated with a SynTC for 30 min at RT. Immunocytochemistry was done following 24-h incubation. Syn10842 (green), total α-Syn polyclonal antibody (rabbit), LB509 (red), total α-Syn monoclonal antibody (mouse), and BIIITubulin (magenta), neuronal marker, were used to stain cells. White arrowheads indicate colocalization of Anti-Syn10842 and Anti-LB509 in neurons. Quantification of average fluorescence intensity of α-Syn aggregates calculated from five different regions of interest (ROI). Bar graph showed as mean ± SD (****p < 0.0001). Scale bar = 10 µm

One of the key phenomena in amyloid strains is that strains act as seeds in the recipient cells, thus recruiting endogenous protein into aggregation and mediating the degeneration of cells [21, 57]. To evaluate the seeding propensity of each α-Syn oligomeric polymorph and the ability of SynTCs to reduce α-Syn propagation in primary neurons, we performed cell-based antibody neutralization assays. This was followed by immunocytochemistry utilizing neuronal marker βIIITubulin (1:1000; Abcam78078), total α-Syn antibody LB509 (1:5000; Abcam 27,766), and total α-Syn rabbit polyclonal antibody Syn10842 (1:1000; Thermo Fisher 10,842–1-AP) to eliminate cross-reactivity with SynTC mouse monoclonal antibodies. We utilized immunocytochemistry to visualize the effects of SynTC neutralization of α-Syn oligomeric polymorphs in primary cortical neurons isolated from mice expressing human α-Syn (Fig. 4). We quantified average fluorescent intensity of α-Syn detected by Syn10842 (Fig. 4c, f, i) and LB509 (Fig. 4b, e, h) to evaluate total α-Syn propagation. SynODA exhibited the highest seeding propensity among the three polymorphs (Fig. 4a, d, g). SynOaCSF (Fig. 4a–c), SynODA (Fig. 4d–f), and SynODHA (Fig. 4g–i) were all differentially immunodepleted by all three SynTCs. SynTC3-immunodepleted neurons exhibiting the highest reduction in total α-Syn. The colocalization profiles for individual regions of interest, specifically neuronal projections from to one cell body to another, were also quantified and results were consistent with these findings (Supp. Fig. 8). We next sought out to determine if cells exposed to toxic α-Syn oligomeric conformers are differentially reduced when immunodepleted by SynTCs.

### SynTCs Differentially Reduce α-Syn Oligomer-Mediated Neurotoxicity

**Fig. 5:**
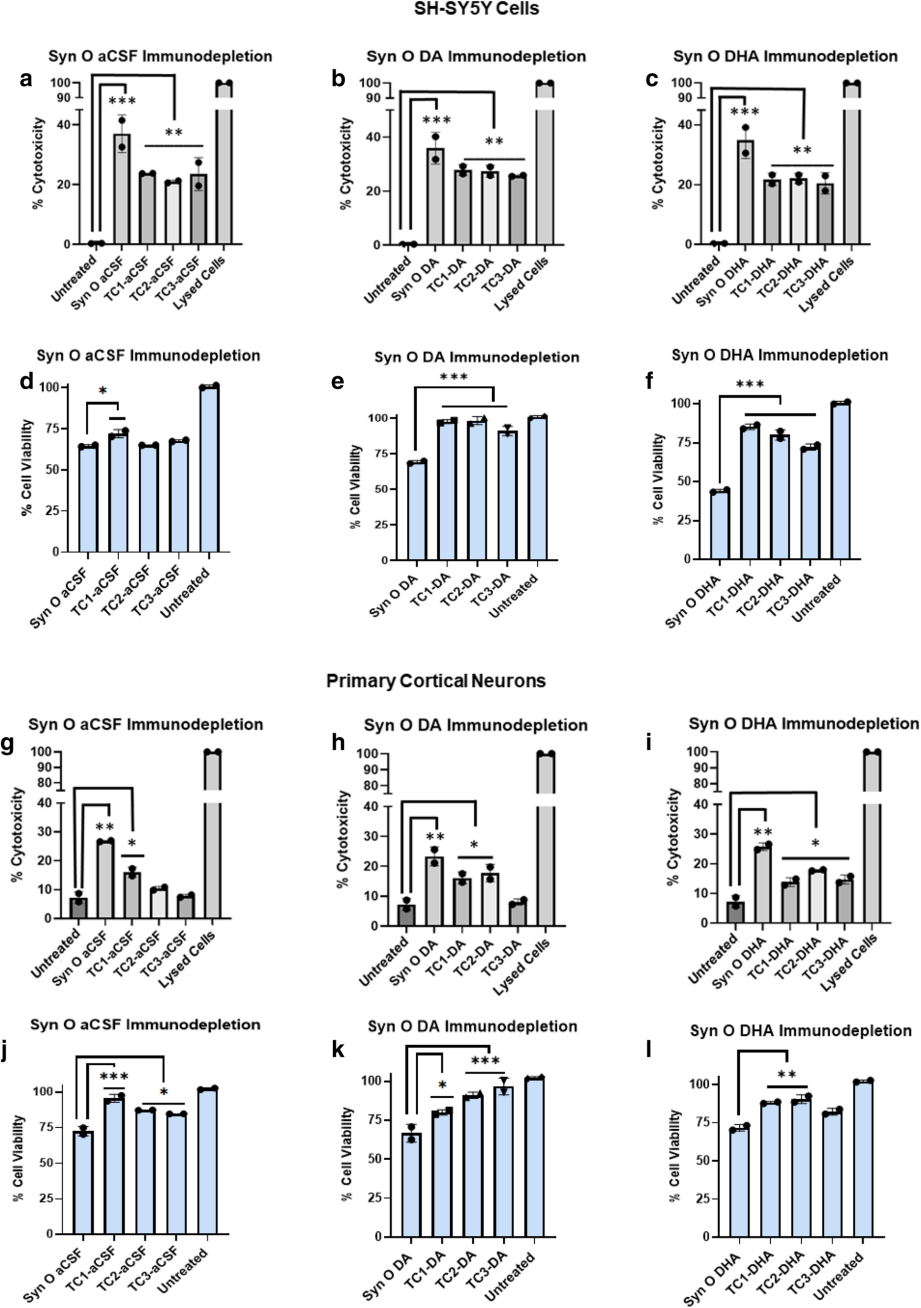
α-Syn oligomeric polymorphs are cytotoxic and are differentially neutralized by SynTCs. SynTCs inhibit cytotoxicity exerted by α-synuclein oligomers in human neuroblastoma SH-SY5Y cells (**a–f**) and primary cortical neurons isolated from mice overexpressing human α-Syn (**g–l**). α-Syn oligomers (0.5uM) were preincubated with a SynTC (2 uM) at a ratio of 1:4 for 30 min at RT and added to the cells for 24 h. Cytotoxicity was analyzed by LDH (gray bars) and MTS (blue bars) cell-based assays. Bars and error bars represent means and standard deviations, respectively (***P < 0.001).

Previous reports observed that exogenously added α-Syn oligomers cause cellular toxicity either by seeding endogenous protein or by acting on cellular membranes [58, 59]. Studies have shown SHSY-5Y cells respond to α-Syn fibrillar seed-induced disruption of protein homeostasis predominantly by secreting α-Syn aggregates [60, 61]. Furthermore, our results reveal that α-Syn oligomeric conformers recruit cytosolic α-Syn aggregates and exhibit distinct seeding potencies and cytotoxicity [21]. We sought to assess the cytotoxic effects of immunodepleting α-Syn oligomeric strains in SH-SY5Y cells (Fig. 5a–f) and primary cortical neurons isolated from mice overexpressing human α-Syn (Fig. 5g–l). Cytotoxicity in both SHSY-5Y cells and primary neurons was measured by LDH (Fig. 5a–c, g–i) and cell viability was measured by MTS cell-based assays (Fig. 5d–f, j–l). α-Syn oligomeric polymorphs exhibit cytotoxicity in SH-SY5Y cell and primary cortical neurons. SynTCs differentially reduce α-Syn oligomer-mediated cytotoxicity in SHSY-5Y cells and primary cortical neurons. SHSY-5Y cells treated with α-Syn oligomeric conformers exhibited higher cytotoxicity than primary neurons. SynTC immunodepletion caused a reduction in cytotoxicity and an increase in cell viability for each oligomeric conformer in both SHSY-5Y cells and primary neurons with varying significance. These results suggest that SynTCs bind in a polymorph-specific manner and that α-Syn oligomeric polymorphs mediate distinct neurotoxicity that can be reduced by immunodepletion.

## Discussion

α-Syn is a 140-amino acid neuronal protein that is the major component of Lewy bodies, a pathological hallmark of synucleinopathies such as Parkinson’s disease, dementia with Lewy bodies, and multiple system atrophy [62, 63]. Investigating the therapeutic targeting of polymorphic α-Syn oligomers and its effects on α-Syn endogenous aggregation and mediated toxicity provides insight into the design and molecular recognition needed for antibodies to effectively target biologically relevant amyloidogenic aggregates [21, 34, 64–67]. The formation of distinct polymorphs of amyloidogenic proteins tau, amyloidβ, and α-Syn presents a new challenge for developing diagnostics and therapeutics [15, 21, 52, 53]. In this study, we employ biochemical and biophysical methods to investigate the biological and immunoreactive properties of three α-Syn oligomeric polymorphs utilizing established methods and three α-Syn Toxic Conformation Monoclonal Antibodies, SynTCs, developed by our lab. Initial epitope mapping data showed SynTC binding sites are discontinuous, nonoverlapping sequence patterns further suggesting conformational epitopes [68].

α-Syn is conformationally dynamic, and this often poses a challenge for developing effective antibodies [69–71]. We utilized dot blotting and western blotting to evaluate protein recognition with both denaturing and non-denaturing conditions. Both immunoblotting conditions in addition to the ELISA results showed differences in α-Syn oligomeric strain immunoreactivity. These differences provide evidence for the biological relevance of polymorph-specific antibody interactions. Vaikath et al. showed the detection of distinct α-Syn micro-aggregates and thin neurites in synucleinopathy brain tissue utilizing conformation-specific antibodies [72]. Furthermore, Choi et al. recently demonstrated α-Syn conformation-specific antibodies promoted phagocytosis of extracellular α-synuclein aggregates [73]. Our study further supports these findings while considering physiological conditions that contribute to the oligomerization and heterogeneity observed in neurodegenerative diseases with α-Syn pathology.

We used highly sensitive isothermal titration calorimetry to characterize the thermodynamic binding interactions between SynTCs and different α-Syn species. Isothermal titration calorimetry provides a label-free method for characterizing biomolecule binding reactions, giving insight to functionality. Antibodies binding in the low nanomolar range (10^−9^) to picomolar (10^−12^) range are considered high-affinity antibodies [74]. Each SynTC exhibited a distinct binding profile to oligomeric α-Syn. SynTC1 and SynTC2 differed in terms of binding and stoichiometry, but still exhibited high affinity binding to oligomeric α-Syn. Our study shows differential binding and entropy in a species-specific manner. While there are minimal antibodies targeting aggregated α-Syn being considered for therapeutic treatment, our study shows the biological relevance of polymorphic aggregates. In addition, studies have shown antibody subtype correlates with differences in thermodynamic binding parameters [75–77]. Syn TC1 consists of a IgG2a/Lambda isotype, while SynTC2 and SynTC3 consist of a IgG1/Kappa isotype, which may explain SynTC1’s high affinity to α-Syn oligomer compared to SynTC2 and SynTC3. Differences in antibody subtype and subclass may affect α-Syn species-specific binding which provides insight to both antibody and species functionality [78–82].

Antibody binding exhibits minor variability due to technique-specific conditions [83, 84]. Therefore, we utilized a combination of different methods to conduct a thorough investigation of α-Syn oligomeric polymorph immunoreactivity and binding. In contrast to western blots, proteins are not electrophoretically separated by size in dot blots. Limitations of this feature include difficult detection of non-specific binding and the method does not give any information regarding molecular weight, an important characteristic for distinct protein aggregates. Western blotting involves separation and denaturation which may affect antigenicity and ultimately antibody reactivity. Providing both immunoblotting techniques accounts for limitations of both techniques. In terms of oligomer versus fibril SynTC selectivity, there is a complex dynamic equilibrium among oligomeric and various soluble and insoluble higher-order oligomers and protofibrils [85]. For this reason, conformation-specific antibodies may prefertially bind aggregated forms of α-Syn [86, 87]. Further studies are required to investigate the effects of this selectivity.

SynTCs did not exhibit binding to α-Syn fibrils in ITC experiments. ITC results exhibit differences compared to immunoblotting and ELISA results, specifically SynTC3 did not exhibit binding to α-Syn species in ITC experiments. Protein aggregation is dynamic, and any conformational changes can impact the measured binding interaction [75, 76]. ITC is conducted in solution; however, oligomers are hydrophobic and bind to surface. Entropic changes due to buffer solution can affect the binding of aggregated α-Syn; therefore, we utilized unmodified α-Syn oligomer [88, 89]. This may explain differences observed in biophysical experiments [48, 90–92]. It has been shown conformational epitopes might be preferred for applications involving protein targets in their native state while linear epitopes might be preferred for applications involving protein denaturation [73]. Commercial total α-Syn antibody, LB509, exhibited increased immunoreactivity to all α-Syn species compared to SynTCs and served as a positive control for all biochemistry experiments. These differences reveal considerations for utilizing conformation-specific antibodies for polymorphic amyloidogenic aggregates. We investigate the effects of these antibodies varied selectivity on cytotoxicity and α-Syn propagation in the cell culture experiments.

Amyloid polymorphs including α-Syn act as seeds in recipient cells and recruit endogenous protein into aggregation ultimately resulting in cell death [21, 57]. To further investigate the effects of polymorphic α-Syn oligomers, we immunodepleted α-Syn oligomeric polymorphs in primary cortical neurons overexpressing human α-Syn and examined changes in α-Syn endogenous aggregation and neurotoxicity. We have shown when α-Syn oligomeric polymorphs are exposed to human neuroblastoma, SH-SY5Y, cells act as potent seeds of α-Syn endogenous aggregation and cytotoxicity in a dose-dependent manner [21]. The selected dose and time of incubation were corroborated by other studies [21, 40, 41, 43]. When we examined the effects of α-Syn oligomeric polymorph immunodepletion in primary neurons, we observed differential reduction of α-Syn endogenous aggregation in a polymorph-specific manner. SynODA and SynODHA were more potent seeds of endogenous aggregation compared to SynOaCSF. Furthermore, neurons treated with SynOaCSF and SynODHA exhibited morphological defects and decreased dendrite growth. While oligomers are the most toxic species, physiological conditions, protein–protein interactions, and posttranslational modifications have been implicated in increased oligomerization and internalization of amyloid oligomers [22–24, 45, 46, 93].

Neurotoxicity is a key pathological process that occurs in synucleinopathies, and there are currently no disease-modifying treatments that can neutralize this cellular toxicity. When α-Syn oligomeric polymorphs are immunodepleted by each SynTC, a reduction in neurotoxicity is observed in primary cortical neurons isolated from mice overexpressing human α-Syn. This reduction in toxicity is also shown in SHSY-5Y cells. Differential reduction of toxicity following SynTC immunodepletion suggests polymorph-specific mediated cytotoxicity. While the significance of differences varies for the assays, the values of toxicity and viability agree for each polymorph. The best way to target conformationally dynamic amyloidogenic aggregates remains unclear; however, antibodies that target oligomeric polymorphisms halt oligomer propagation and reduce neurotoxicity that may be important for inducing a protective response to protein aggregation [34, 94]. Further characterization utilizing in vivo models is needed to investigate the immunotherapeutic potential of α-Syn oligomeric polymorphisms and their role in the pathological implications and behavior phenotypes observed in synucleinopathies [34, 47, 64, 65, 95]. In vitro studies in this manuscript lay the foundation for our investigation into the role of polymorphic oligomers and their theorized correlation with the distinct pathology and behavioral symptoms reflected in Parkinson’s disease, dementia with Lewy bodies, and Alzheimer’s disease.

Identifying the biological properties of amyloidogenic polymorphs is essential to improving the design of immunotherapeutic approaches targeting conformationally distinct proteinaceous aggregates. Amyloidogenic polymorphisms may reflect the variability in behavioral phenotypes and pathological implications observed in neurodegenerative diseases [37, 96]. While multiple antibodies that target α-Syn are being developed and investigated, these antibodies target all forms of α-Syn rather than specific aggregates or polymorphisms of α-Syn aggregates [37– 39]. Overlapping proteinopathies have been observed in multiple neurodegenerative diseases and oligomeric polymorphisms may contribute to these protein–protein interactions [21, 40, 41, 97, 98]. It is important to further optimize the efficacy of utilizing antibodies to target the conformational heterogeneity of amyloidogenic oligomers. These findings will further improve the future of novel neurodegenerative disease therapeutics.

## Supplementary Information

Below is the link to the electronic supplementary material.

Supplementary file1 (DOCX 13919 KB)

## Data Availability

The datasets generated during and/or analyzed during the current study are available from the corresponding author on reasonable request.

## Abbreviations

DLB: Dementia with Lewy bodies
PD: Parkinson’s disease
α-Syn: Alpha-synuclein
SynO: α-Syn oligomer
aCSF: Artificial cerebrospinal fluid
DA: Dopamine
DHA: Docosahexaenoic acid
SynTCs: α-Syn toxic conformation monoclonal antibodies
ELISA: Enzyme-linked immunosorbent assay
ITC: Isothermal titration calorimetry
LDH: Lactate dehydrogenase
MTT: 3-(4,5-Dimethylthiazol-2-yl)-2,5-diphenyltetrazolium
hSyn: Human α-Syn
ICC: Immunocytochemistry
