# Supplementary material for: Polymorphic Alpha-Synuclein Oligomers: Characterization and Differential Detection with Novel Corresponding Antibodies
Source: Mol Neurobiol. 2023 Jan 28;60(5):2691–705. doi: 10.1007/s12035-023-03211-3 (PMC9883140; doi:10.1007/s12035-023-03211-3)
Supplement: Supplementary file 1 — Supplementary file1 (DOCX 13919 KB) [file 12035_2023_3211_MOESM1_ESM.docx]

***Supplemental Figure 1. Dot blot quantification.*** *α-Syn monomer, different α-Syn oligomer preparations, α-Syn fibrils, and amyloidogenic proteins, tau and amyloid β were characterized by dot blotting with primary antibodies: SynTC1 (****a****), SynTC2 (****b****), SynTC3 (****c****). Dot blot quantification reveals the immunoreactivity and selectivity of the SynTCs for α-synuclein, confirmed by total Syn antibody, LB509 (****d****).*

*
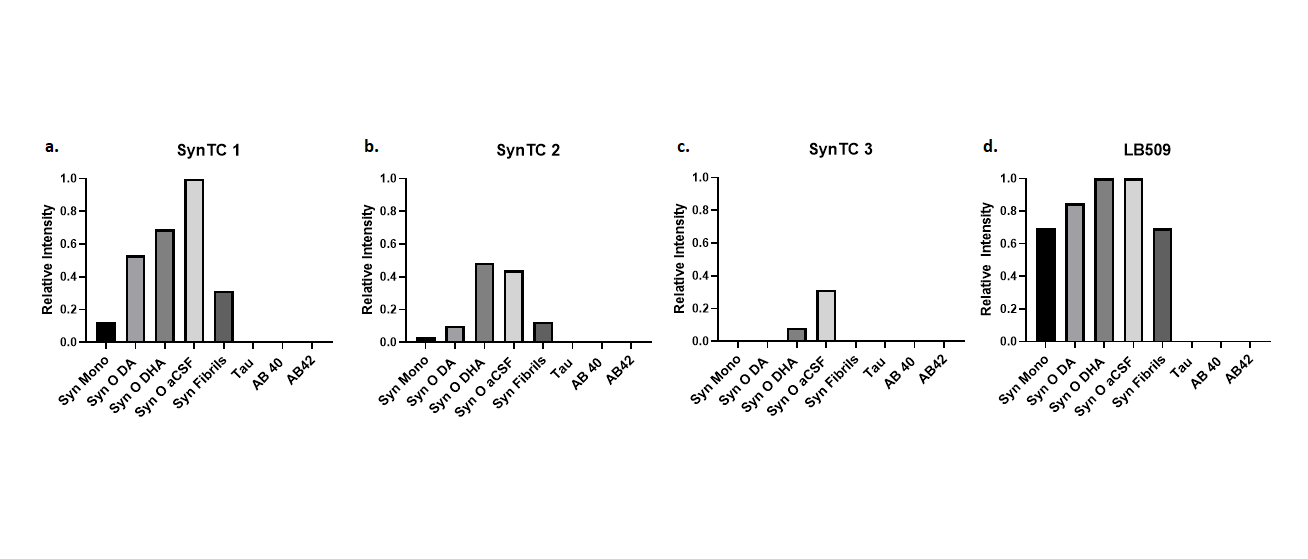
*

*
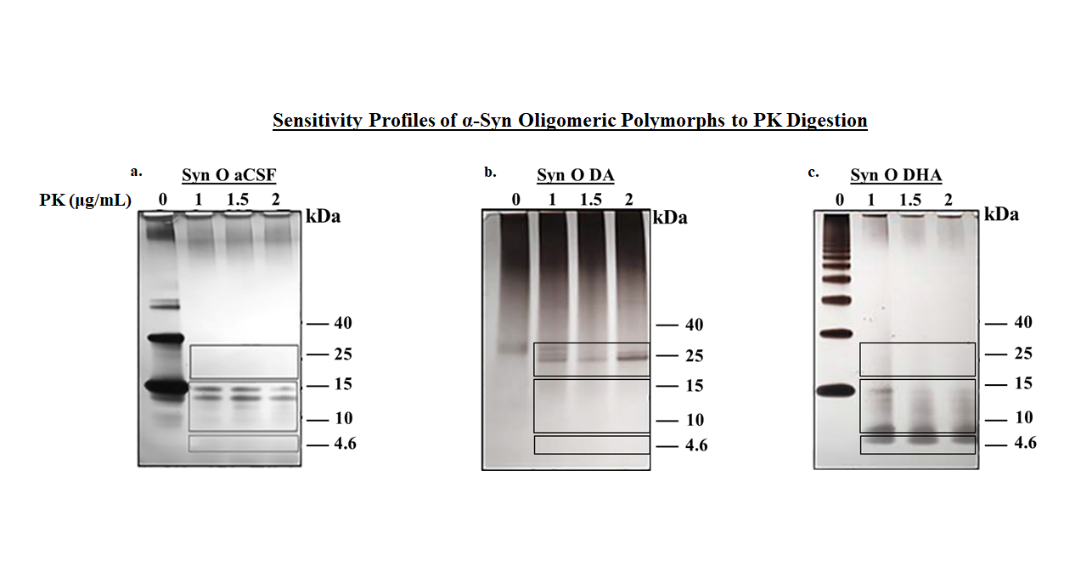
*

**Supplemental Figure 2.** Proteolytic digestion profiles of α-Syn oligomeric polymorphs. Silver staining images of α-Syn oligomers, Syn O aCSF (a), Syn O DA (b), Syn O DHA (c) digested with 1, 1.5, and 2 μg/mL proteinase K (PK) enzyme.


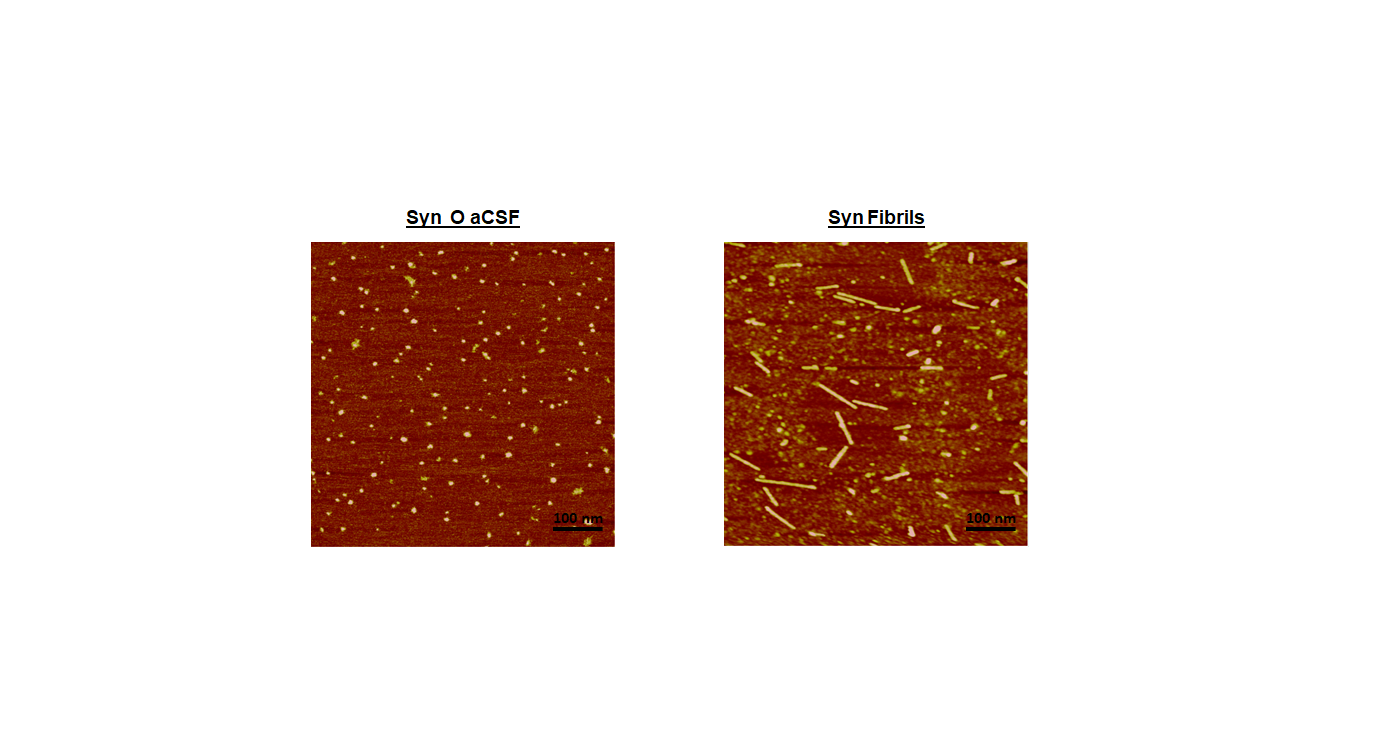


**Supplemental Figure 3. Atomic Force Microscopy of Syn O aCSF and Syn Fibrils.** Representative AFM images of Syn O aCSF and Syn Fibrils. Syn O aCSF exhibit spherical structures while Syn Fibrils exhibit protofilaments.

**
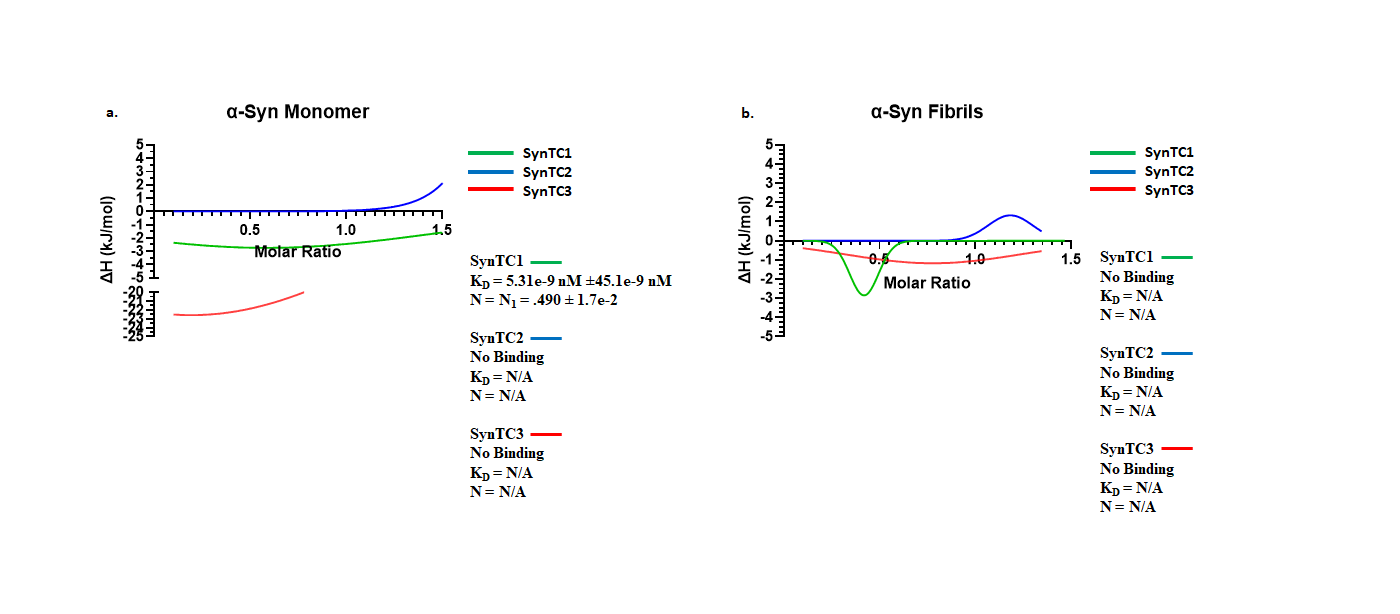
**

**Supplemental Figure 4. Isothermal Titration Calorimetry (ITC) Binding profiles of SynTCs and monomeric and fibrillar α-Syn.** Integrated binding curves of the isothermal titration calorimetry (ITC) experiment of 2 μM α-Syn monomer (a) or fibrils (b) titrated with 8 μM SynTC at 25 °C. The binding curve of each experiment fitted with an n independent binding site model is shown in the graph. Thermodynamic and stoichiometric parameters obtained from the fitting of the binding curve are shown.


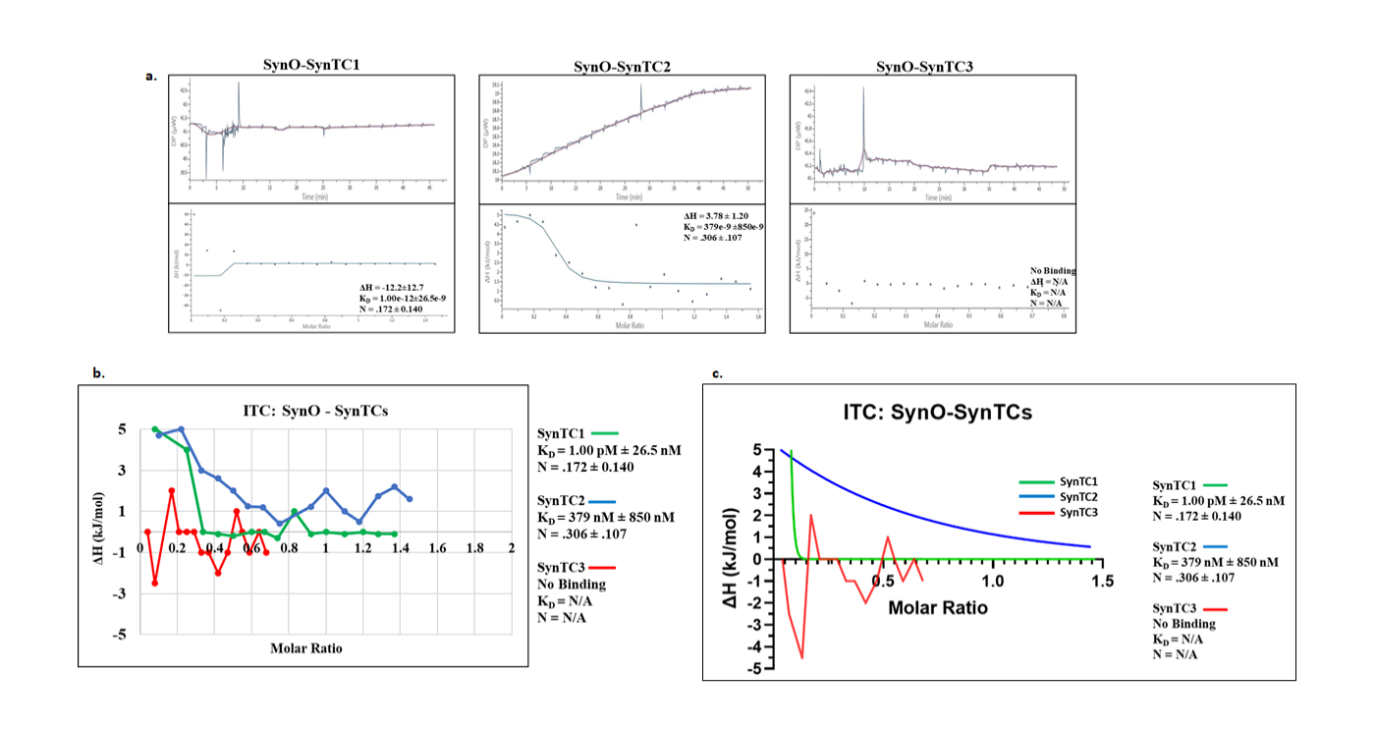


**Supplemental Figure 5. Fitting of α-Syn oligomer:SynTC ITC data.** Primary ITC injection data generated through MicroCal PEAQ-ITC analysis software (**a**). Outliers were determined and excluded utilizing analysis software. Data points were then extracted and graphed in GraphPad (**b**). A Gaussian nonlinear fit model was used to fit the integrated binding curves utilizing GraphPad (**c**).


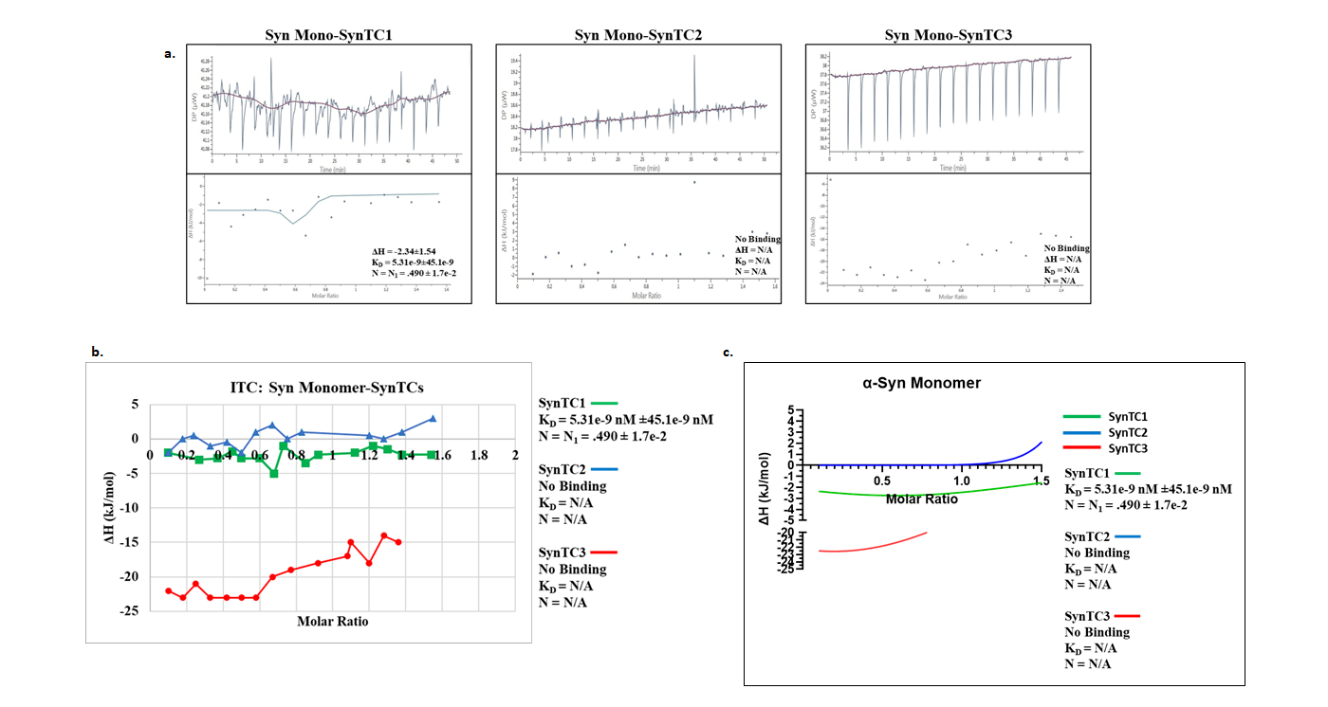


**Supplemental Figure 6. Fitting of α-Syn monomer:SynTC ITC data.** Primary ITC injection data generated through MicroCal PEAQ-ITC analysis software (**a**). Outliers were determined and excluded utilizing analysis software. Data points were then extracted and graphed in GraphPad (**b**). A Gaussian nonlinear fit model was used to fit the integrated binding curves utilizing GraphPad (**c**).


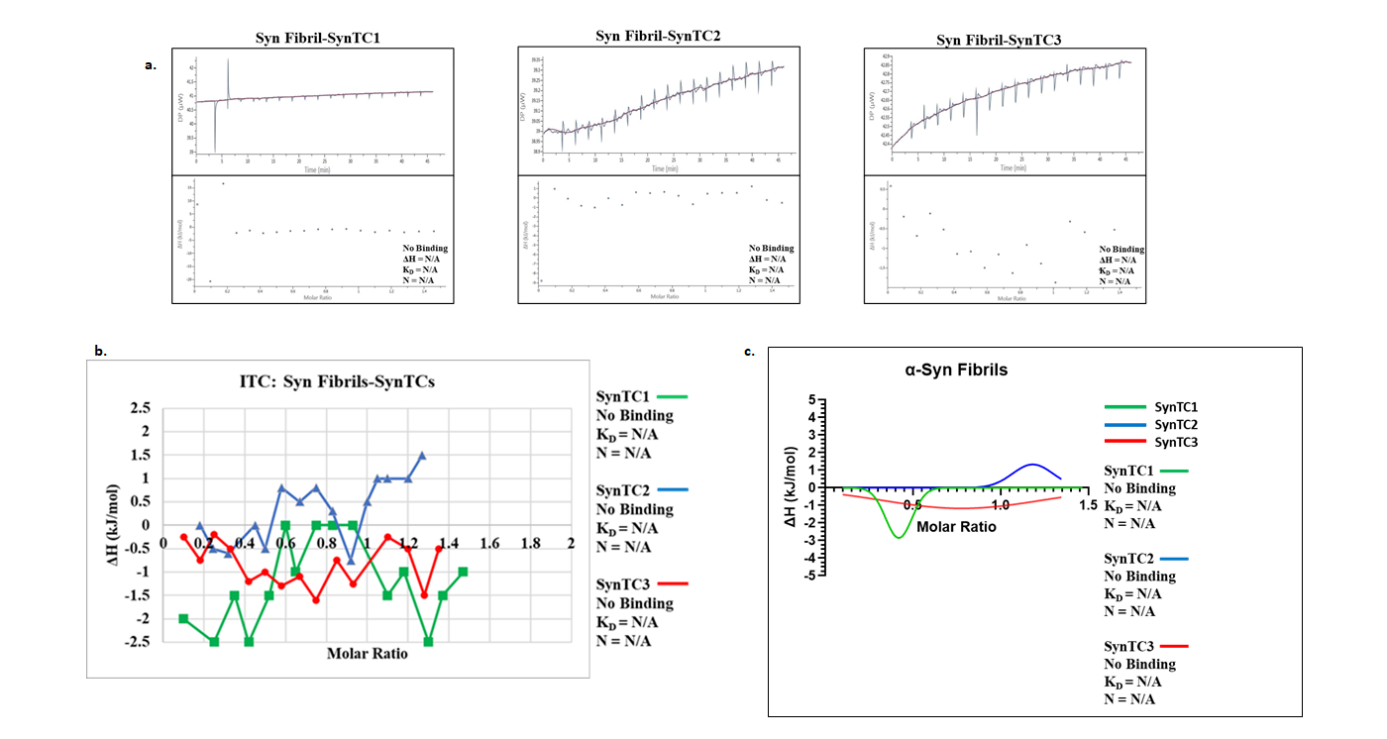


**Supplemental Figure 7. Fitting of α-Syn monomer:SynTC ITC data.** Primary ITC injection data generated through MicroCal PEAQ-ITC analysis software (**a**). Outliers were determined and excluded utilizing analysis software. Data points were then extracted and graphed in GraphPad (**b**). A Gaussian nonlinear fit model was used to fit the integrated binding curves utilizing GraphPad (**c**).


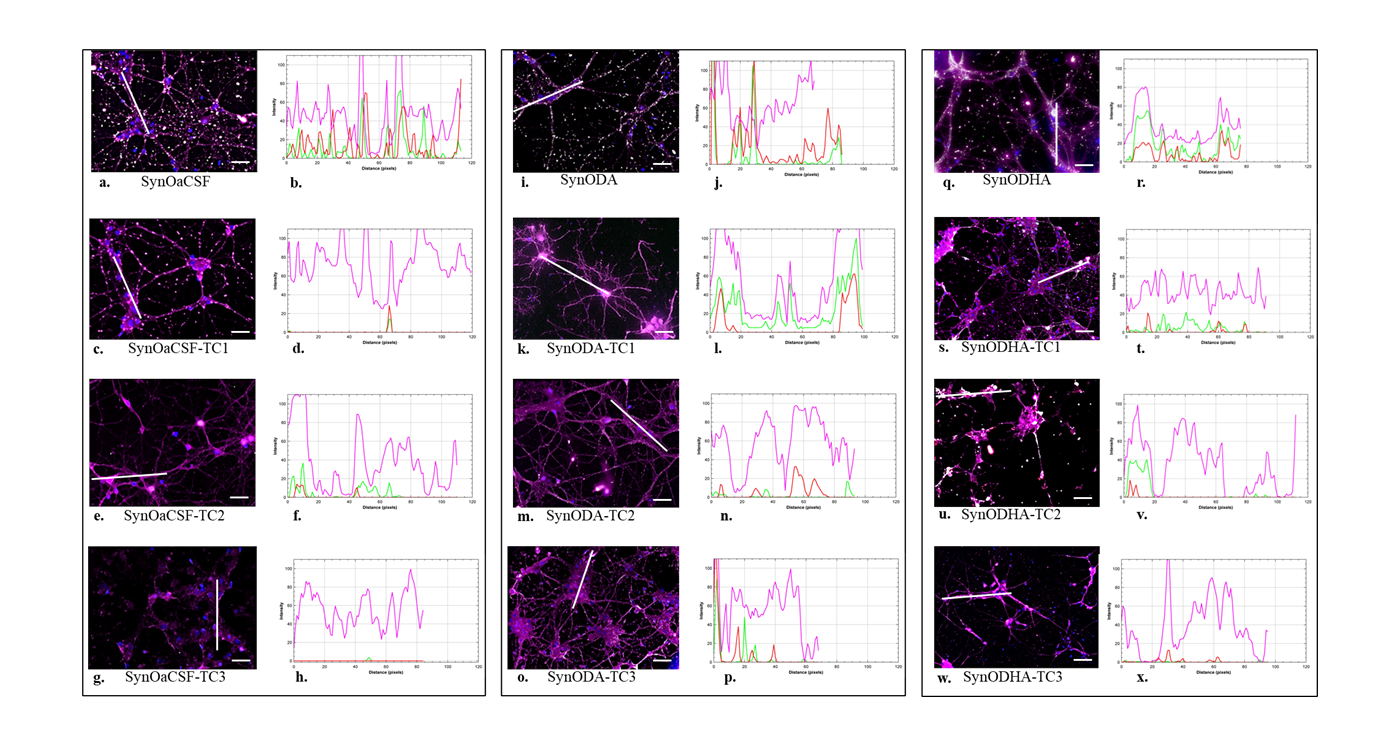


**Supplemental Figure 8. SynTC neutralization of α-Syn oligomeric polymorphs affect α-Syn endogenous aggregation.** Primary cortical neurons were treated with α-Syn oligomer or α-Syn oligomer preincubated with a SynTC for 30 mins at RT. Immunocytochemistry was done following 24hr incubation. Syn10842 (green), total α-Syn polyclonal antibody (rabbit), LB509 (red), total α-Syn monoclonal antibody (mouse), and BIIITubulin (magenta), neuronal marker, primary antibodies were used to stain cells. The colocalization profiles for individual selected neurons are shown above. Region of interest (ROI) marked in white line indicating colocalization of anti-Syn LB509, anti-Syn10842, and anti-BIITubulin in neurons.
